# Parapharyngeal Space Angiofibroma: A Diagnostic Surprise

**DOI:** 10.22038/ijorl.2020.40983.2561

**Published:** 2021-03

**Authors:** Naresh K Panda, Sourabha K Patro, Samarendra Behera, Parimal Agarwal, Ashim Das

**Affiliations:** 1 *Department of Otolaryngology and Head and Neck Surgery, PGIMER, Chandigarh, India.*; 2 *Department of Histopathology, PGIMER, Chandigarh, India.*

**Keywords:** Angiofibroma, Nasopharyngeal Neoplasms, Parapharyngeal Space, Parapharyngeal Angiofibroma

## Abstract

**Introduction:**

Angiofibromas classically develop in the lateral wall of the nasopharynx from the sphenopalatine region. Extra-nasopharyngeal angiofibromas are rare entities, with the maxillary sinus being the most common site. Parapharyngeal angiofibroma is an extremely rare entity, being seldom reported in English literature.

**Case Report:**

We present a case of parapharyngeal angiofibroma, which came as a diagnostic surprise in a young adult 25-year-old male. The radiological picture showed a highly vascular lesion, which did draw our attention for not going for a direct or guided FNAC, and upfront excision was planned through the transcervical route. A firm 5 * 7 cm mass was excised and sent for histopathologic examination. The histopathology showed angiofibroma like features as a diagnostic surprise as angiofibroma of parapharyngeal space is a known but rare entity.

**Conclusion:**

The knowledge of angiofibroma as a differential in parapharyngeal space will help the clinicians to properly deal with these disorders during preoperative evaluation and definitive surgery and thus prevent the chances of vascular injuries and complications associated with an FNAC. A high level of suspicion regarding this differential as a possible lesion in the parapharynx is required for the entity's diagnosis.

## Introduction

Angiofibromas are classically seen in the nasopharynx. Extra-nasopharyngeal angio- fibromas have rarely been reported. Primary sites of extra-nasopharyngeal angiofibromas are mentioned to be the nasal septum, maxillary sinus, and ethmoids (1-6). 

Parapharyngeal angiofibroma is an extremely rare entity and is a diagnostic surprise for the clinician. A high level of suspicion is required for consideration of angiofibroma as a differential diagnosis of parapharyngeal space (PPS) lesions. We report a case of extra-nasopharyngeal angiofibroma in a young male. 

## Case Report

A 25-year gentleman presented complaining gradual change in voice quality, heaviness of throat, and occasional swallowing difficulty for the last two years without definite dysphagia, dyspnea, or odynophagia. History pertaining to other systems and addiction history was negative. 

**Fig1:**
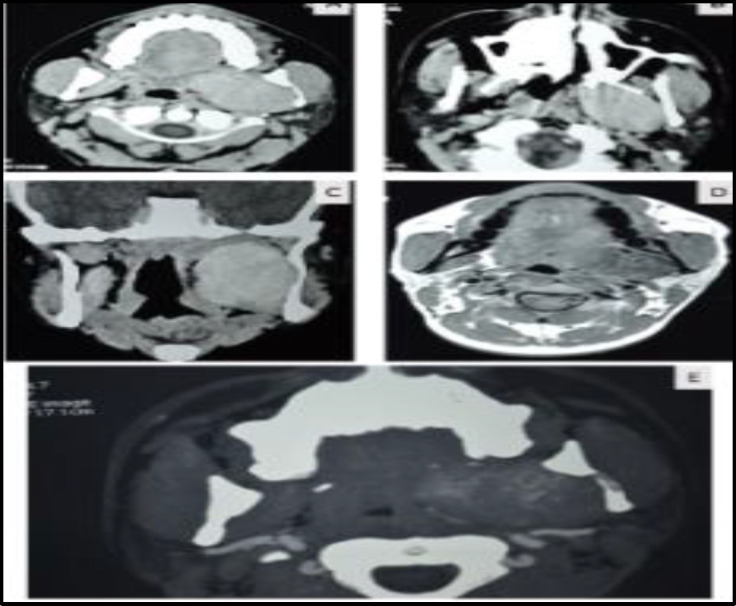
**A** **)** Axial contrast enhanced computerized image showing diffusely enhancing smooth bordered mass in left parapharyngeal space. **B:** Axial Image Diffusely enhancing mass in the superior part of parapharynx splaying the lateral and medial pterygoid plates. **C)** Coronal image showing a highly enhancing homogenous mass in left parapharyngeal space extending superiorly till skull base and inferiorly till the lower border of mandible. **D)** Axial magnetic resonance image showing the mass in the parapharyngeal spaces having a heterogeneous picture with many flowvoids seen with in the lesion. **E)** Axial computerized angiographic tomography (CT –Angio) image showing blood vessels with the lesion present in left parapharyngeal space anterior to both the carotids

Head and neck examination revealed a left lateral pharyngeal wall bulge with medially pushed tonsil. Visible or palpable neck swelling or retro mandibular fullness were absent. It was not bimanually palpable. Intraoral palpation revealed non-pulsatile soft to firm swelling with restricted mobility. Imaging studies revealed a hyperdense soft tissue mass lesion involving the pre-styloid compartment of left PPS between the root of pterygoids anteriorly, styloid process posteriorly, and body of mandible laterally with posteriorly displaced great vessels (Fig.1A-D); medially bulged lateral wall from nasopharynx superiorly to hypopharynx inferiorly; anteriorly deviated lateral pterygoid plate by occupying the retro pterygoid space between the pterygoid plates; and diffusely enhancing on contrast administration. 

CT angiographic images revealed a highly vascular mass with multiple vessels with posteriorly pushed carotids (Fig.1E). 

**Fig 2:**
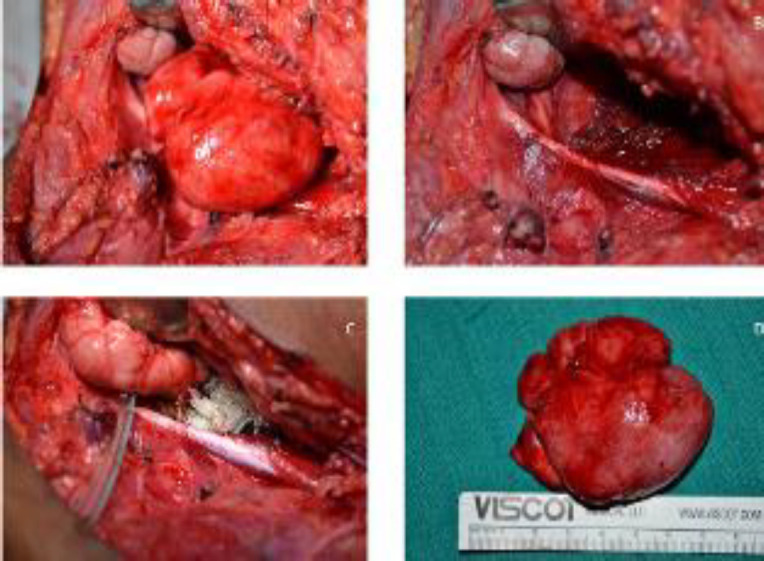
**A** **)** Picture of the para-pharyngeal space tumor of firm consistency popping out after exposure. **B)** Bed of the tumor after removal of the tumor. **C)** Surgical Site after hemostasis. **D)** Size of the tumor after removal

A decision to avoid fine-needle aspiration cytology (FNAC) examination was taken considering the vascular nature of the lesion seen in angiography, and surgical excision was planned. Trans-cervical excision with horizontal lateral incision was planned, and mandibulotomy was reserved as an optional approach to increase exposure at the time of need. A 5 * 7 cm firm swelling involving the para pharyngeal space was found and dissected out. (Fig.2 A-D)

**Fig 3:**
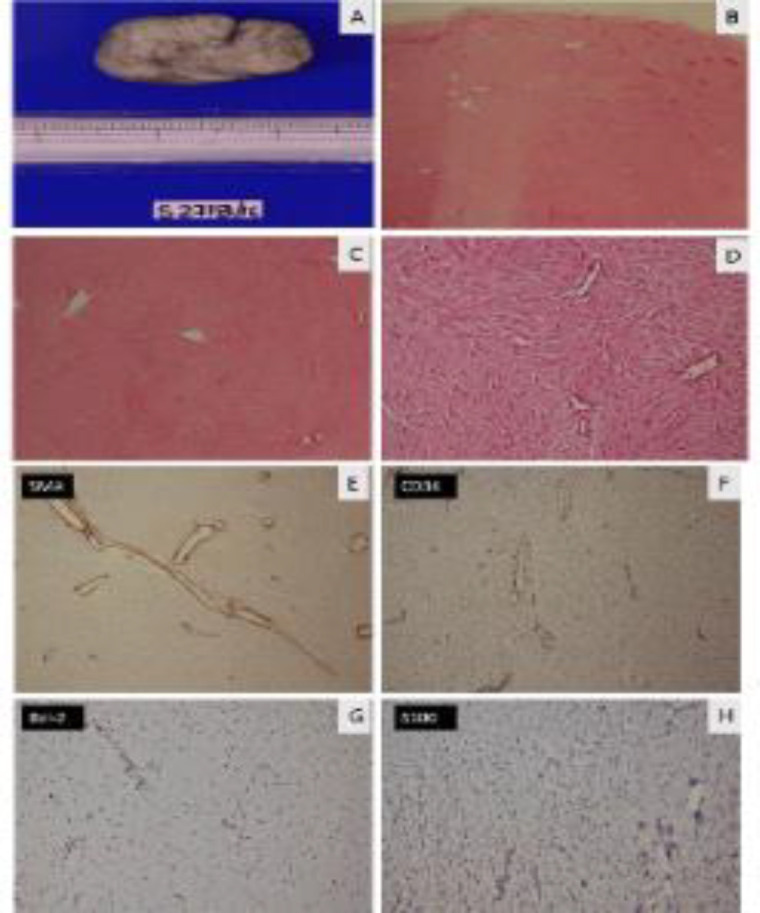
**A** **)** Cut surface of the tumor showing greyish white fibrotic stroma. **B)** Well circumscribed pauci cellular lesion (H&E x 20).**C:** Numerous scattered slit like and stag horn type blood vessels (H&E x 40). **D)** Collagenous pauci cellular stroma composed of bland spindle and stellate fibroblasts surrounding the blood vessels lacking an elastic lamina (H&E x 200) **E)** IHC for SMA - Pericytes in blood vessels (Immunoperoxidase x 100). **F)** IHC for CD34 - Negative in fibroblasts however highlighted the blood vessels (Immunoperoxidase x 100). **G)** IHC for Bcl-2 - Nuclear positivity in few scattered fibroblasts (Immunoperoxidase x 100). **H)** IHC for S100 - Negative (Immunoperoxidase x 100)

The postoperative gross evaluation revealed a well-circumscribed greyish-white mass with a firm fibrotic cut surface (Fig.3A). Microscopic examination revealed a well-circumscribed pauci-cellular lesion (Fig.3B). Higher magnification showed numerous scattered slit-like and staghorn type blood vessels within the fibrotic pauci-cellular stroma of the tumor (Fig.3C). Higher magnification revealed collagenous pauci-cellular stroma composed of bland spindle and stellate fibroblasts surrounding the blood vessels lacking an elastic lamina (Fig.3D). This histopathology was not corroborating with any of the commonly found parapharyngeal tumors, which led us for immunohistochemistry studies with SMA, CD 34, BCL-2, and S-100. IHC for SMA showed the pericytes in blood vessels (Fig.3E). IHC for CD34 (Fig.3F) was negative in fibroblasts while it had highlighted the blood vessels. IHC for Bcl-2 (Fig. 3G) showed nuclear positivity in a few scattered fibroblasts; however, IHC for S100 (Fig.3H) was negative. A final diagnosis of parapharyngeal space (PPS) angiofibroma was made. 

## Discussion

Angiofibroma is a fibrovascular tumor of nasopharynx seen in young adolescent males accounting for <0.5% of the head and neck tumors (5). It most commonly and almost exclusively arises from the sphenopalatine foramen in the lateral nasopharyngeal wall. Few cases of extra-nasopharyngeal angiofibroma have been reported in the literature. Maxillary sinus is the most common site of extra-nasopharyngeal angiofibromas (7) with sporadic reports of non-nasal/paranasal sites such as hypopharynx and larynx (8,9).

Extra-nasopharyngeal angiofibromas are common in females with older age of presentation compared to the nasopharyngeal angiofibromas (10). Literature review revealed rare instances of parapharyngeal angiofibromas and rare reports of other variants of angiofibroma such as giant cell angiofibroma (6,11,12). There have been various theories described in the literature in almost the last half-century to explain the development of angiofibromas. Still, none of these could have been proved with enough evidences (13-15).

Currently, the most accepted theories of its development include testosterone dependent vascular malformations arising from discontinuous vascular basal laminae having focal lack of pericytes, with the irregularity of smooth muscle layers, developing from the persistent fibrovascular nidus in and around the nasopharynx with molecular evidence favoring these (13,16-18). 

As these nidus are also present in the various junction of skull bones and parapharynx and the lateral side of pterygoids and parapharynx, it is postulated that parapharyngeal angiofibromas and extra nasopharyngeal angiofibromas might also be arising by such ways just as nasopharyngeal angiofibromas, however clear evidence for or against any argument regarding the development of extra nasopharyngeal angiofibromas are lacking in literature (5), because of the extreme rarity of the tumor. 

Surgical excision is the preferred treatment for angiofibromas, with radiotherapy being considered only for unresectable cases. Extra-nasopharyngeal angiofibromas have a better postoperative prognosis due to the difficulties in surgical exposure and technical expertise involved in surgical excisions of nasopharyngeal angiofibromas (8). Highly vascular nature, absence of smooth muscles around blood vessels and extremely fibrous nature increases chance of threatening hemorrhage if FNAC or biopsy is attempted. Hence, high suspicion is required to avoid catastrophes in cases of nasopharyngeal angiofibromas, and attempts towards tissue diagnosis are considered relative contraindications in such cases. The same also holds true for preoperative tissue diagnosis of radiologically suspected non-nasopharyngeal angiofibromas, like this case, to a great extent, as described in the literature (5). Hence, in our institute and country, tissue diagnosis attempts are considered a relative contraindication for the neck's suspected vascular neck tumors such as carotid body tumors, paragangliomas, and extra nasopharyngeal angiofibromas. This tumor had a highly vascular contrast-enhancing computerized tomographic appearance; hence FNAC was avoided, and surgical excision was planned with a working diagnosis of paraganglioma. Other clinical differentials for this case were minor salivary gland tumor, neurogenic tumor, and solitary fibrous tumor. Postoperative histopathologic appearance and diagnosis was a surprise. The microscopic picture helped in making the diagnosis, which showed two components as a pauci cellular collagenous stroma containing bland spindle and stellate shaped fibroblasts (highlighted on Bcl-2 IHC, which is also a fibroblast marker just like CD34) and scattered slit-like and staghorn blood vessels lacking an elastic layer just like its nasopharyngeal counterpart. SMA negativity in stromal cells ruled out a possibility of hemangiopericytoma. S100 negativity ruled out schwannoma or a neurofibroma. Hence the diagnosis of angiofibroma of parapharyngeal space was made in this case, considering the histology and corroborating it with the preoperative radiologic investigations. 

## Conclusion 

In the present case, the surgical excision was complete and postoperative diagnosis was a surprise. Parapharyngeal angiofibroma is a rare diagnosis. However, the knowledge of its possibility and suspicion helps the surgeon prevent inadvertent complications. 

## References

[1] Mohindra S, Grover G, Bal AK (2009). Extranasopharyngeal angiofibroma of the nasal septum: a case report. Ear, nose, & throat journal..

[2] Mutlu V (2015). Angiofibroma from the tail of the inferior turbinate. The Eurasian journal of medicine..

[3] Handa KK, Kumar A, Singh MK, Chhabra AH (2001). Extranasopharyngeal angiofibroma arising from the nasal septum. International journal of pediatric otorhinolaryngology..

[4] Bhagat S, Verma RK, Panda NK (2011). Extranasopharyneal angiofibroma in an adult: a rare presentation. Indian journal of otolaryngology and head and neck surgery..

[5] Windfuhr JP, Remmert S (2004). Extranasopharyngeal angiofibroma: etiology, incidence and management. Acta oto-laryngologica..

[6] Lee BH (2015). Parapharyngeal Angiofibroma: A Case Report. Iranian journal of radiology..

[7] Panesar J, Vadgama B, Rogers G, Ramsay AD, Hartley BJ (2004). Juvenile angiofibroma of the maxillary sinus. Rhinology..

[8] Hsieh ST, Guo YC, Tsai TL, Chen WY, Huang JL (2004). Angiofibroma of the hypopharynx. Journal of the Chinese Medical Association..

[9] Renukananda GS, Basavaraja PK, Naik AS, Maheshwari M, Balaji NK, Thangavelu G (2008). Atypical angiofibroma of larynx - a case report. Indian journal of otolaryngology and head and neck surgery..

[10] Huang RY, Damrose EJ, Blackwell KE, Cohen AN, Calcaterra TC (2000). Extranasopharyngeal angiofibroma. International journal of pediatric otorhinolaryngology..

[11] Johnson JE, Yang PJ, Koopmann CF Jr, Heffner DK (1987). Parapharyngeal angiofibroma. American journal of neuroradiology.

[12] Gonzalez-Perez LM, Sanchez-Gallego F, Haro-Luna JJ, Infante-Cossio P (2010). Giant cell angiofibroma of parapharyngeal space: a report of a new location for a rare tumour. International journal of oral and maxillofacial surgery..

[13] Li W, Ni Y, Lu H, Hu L, Wang D (2019). Current perspectives on the origin theory of juvenile nasopharyngeal angiofibroma. Discovery medicine..

[14] Schlauder SM, Knapp C, Steffensen TS, Bui MM (2009). Aromatase may play a critical role in the pathogenesis of juvenile nasopharyngeal angiofibroma. Fetal and pediatric pathology..

[15] Schiff M (1959). Juvenile nasopharyngeal angiofibroma. a theory of pathogenesis. Laryngoscope..

[16] Starlinger V, Wendler O, Gramann M, Schick B (2007). Laminin expression in juvenile angiofibroma indicates vessel's early developmental stage. Acta oto-laryngologica..

[17] Renkonen S, Hayry V, Heikkila P, Leivo I, Haglund C, Mäkitie AA (2011). Stem cell-related proteins C-KIT, C-MYC and BMI-1 in juvenile nasopharyngeal angiofibroma--do they have a role?. Virchows Archiv.

[18] Doody J, Adil EA, Trenor CC, Cunningham MJ (2019). The Genetic and Molecular Determinants of Juvenile Nasopharyngeal Angiofibroma: A Systematic Review. The Annals of otology, rhinology, and laryngology..

